# PDK4-mediated metabolic reprogramming is a potential therapeutic target for neovascular age-related macular degeneration

**DOI:** 10.1038/s41419-024-06968-0

**Published:** 2024-08-09

**Authors:** Juhee Kim, Yujin Jeon, Jinyoung Son, Haushabhau S. Pagire, Suvarna H. Pagire, Jin Hee Ahn, Akiyoshi Uemura, In-Kyu Lee, Sungmi Park, Dong Ho Park

**Affiliations:** 1 Department of Ophthalmology, School of Medicine, Kyungpook National University, Kyungpook National University Hospital, Daegu, Republic of Korea; 2 Kyungpook National University Cell & Matrix Research Institute, Daegu, Republic of Korea; 3 Department of Biomedical Science, The Graduate School, Kyungpook National University, Daegu, Republic of Korea; 4 BK21 FOUR KNU Convergence Educational Program of Biomedical Sciences for Creative Future Talents, Daegu, Republic of Korea; 5 Department of Chemistry, Gwangju Institute of Science and Technology, Gwangju, Republic of Korea; 6 R&D center, JD Bioscience Inc, Gwangju, Republic of Korea; 7 Department of Ophthalmology and Visual Science, Nagoya City University Graduate School of Medical Sciences, Nagoya, Japan; 8 Department of Internal Medicine, School of Medicine, Kyungpook National University, Kyungpook National University Hospital, Daegu, Republic of Korea

**Keywords:** Metabolic disorders, Diseases of the nervous system

## Abstract

Age-related macular degeneration (AMD) causes severe blindness in the elderly due to choroidal neovascularization (CNV), which results from the dysfunction of the retinal pigment epithelium (RPE). While normal RPE depends exclusively on mitochondrial oxidative phosphorylation for energy production, the inflammatory conditions associated with metabolic reprogramming of the RPE play a pivotal role in CNV. Although mitochondrial pyruvate dehydrogenase kinase (PDK) is a central node of energy metabolism, its role in the development of CNV in neovascular AMD has not been investigated. In the present study, we used a laser-induced CNV mouse model to evaluate the effects of *Pdk4* gene ablation and treatment with pan-PDK or specific PDK4 inhibitors on fluorescein angiography and CNV lesion area. Among PDK isoforms, only PDK4 was upregulated in the RPE of laser-induced CNV mice, and *Pdk4* gene ablation attenuated CNV. Next, we evaluated mitochondrial changes mediated by *PDK1-4* inhibition using siRNA or PDK inhibitors in inflammatory cytokine mixture (ICM)-treated primary human RPE (hRPE) cells. *PDK4* silencing only in ICM-treated hRPE cells restored mitochondrial respiration and reduced inflammatory cytokine secretion. Likewise, GM10395, a specific PDK4 inhibitor, restored oxidative phosphorylation and decreased ICM-induced upregulation of inflammatory cytokine secretion. In a laser-induced CNV mouse model, GM10395 significantly alleviated CNV. Taken together, we demonstrate that specific PDK4 inhibition could be a therapeutic strategy for neovascular AMD by preventing mitochondrial metabolic reprogramming in the RPE under inflammatory conditions.

## Introduction

Age-related macular degeneration (AMD) is a leading cause of blindness among the elderly population [1]. Particularly, neovascular AMD exhibits characteristic choroidal neovascularization (CNV), in which abnormal new vessels penetrate through retinal pigment epithelium (RPE) into the outer retina, compromising vision [2]. Although intravitreal injections of anti-vascular endothelial growth factor (VEGF) antibodies are currently used to treat CNV, more than half of patients have an inadequate response to the treatment despite monthly injections [3]. Furthermore, the current therapeutics require repeated injections which can cause serious infections complications including endophthalmitis [4]. These limitations emphasize the critical need to develop alternative therapeutic targets for CNV.

Because the RPE phagocytoses and degrades shed outer segments throughout life, RPE cells require a significant energy supply that relies almost exclusively on mitochondria, primarily oxidative phosphorylation (OXPHOS) [5–7]. However, under hypoxic or inflammatory conditions, metabolic reprogramming towards aerobic glycolysis is mainly regulated by stabilization of hypoxia-inducible factor 1-alpha (HIF-1*α*), and the metabolic shift results in releasing proinflammatory cytokines [8]. A proinflammatory RPE environment promotes the development of AMD [9]. Furthermore, prior studies of human donors with AMD support that defective RPE mitochondrial function drives AMD pathology [10]. In primary cultures from AMD donors, ATP generation through OXPHOS is decreased, while ATP generation through glycolysis is increased [11].

The mitochondrial pyruvate dehydrogenase complex (PDC) is a central metabolic node mediating pyruvate oxidation, the critical step in OXPHOS. The PDC is regulated by post-translational subunit modifications, including phosphorylation of the E1α subunit of pyruvate dehydrogenase (PDHE1α) by pyruvate dehydrogenase kinase (PDK) 1–4 and de-phosphorylation regulated by pyruvate dehydrogenase phosphatase 1–2 [12]. Prior studies have reported that fursultiamine, a mitochondrial PDH cofactor, alleviated CNV by modulating the RPE metabolic and inflammatory response [13]. Similarly, PDK inhibition, which increases PDC activity, has been used as a therapeutic approach for inflammatory bowel disease [14]. However, the effects of PDK inhibition on metabolism processes in the RPE have not been investigated. Thus, in this study, we investigated whether PDK inhibition, particularly with small-molecule PDK4 inhibitors, alleviates CNV by regulating RPE metabolic and inflammatory reactions.

## Materials and methods

### Animal studies

Animal experiments were conducted in accordance with the guidelines by the Association for Research in Vision and Ophthalmology Statement for the Use of Animals in Ophthalmic and Vision Research. Animal Care Committee of the Kyungpook National University (No. 2019-0104-1) approved the animal studies. *Pdk4* knockout mice (*Pdk4*^−/−^) were bred in an in-house animal facility [15].

### Laser-induced CNV model and treatment

Animals were randomly assigned to one of experimental or control group (5 mice/group). Seven days before CNV induction, 7-week-old male C57BL/6J mice were treated with dichloroacetate (DCA 250 mg/kg in saline, Sigma-Aldrich, St. Louis, MO, USA) or GM10395 (1 mg/kg; 10 mg/mL in 1% DMSO), or normal saline by oral gavage daily for 14 days. A 532-nm OcuLight GLx Laser System (IRIDEX Corporation, Mountain View, CA, USA) was used to generate 4–10 CNV lesions in mice with the parameters as described previously [16, 17]. Photocoagulation spots with hemorrhage or no bubble formation at the laser site were excluded. Seven days after CNV induction, pigmented RPE and choroid tissues (RPE/choroid) were dissected from the above transparent tissues (retina tissue; from nerve fiber layer to photoreceptor layer) and collected for choroidal flat mount and protein or RNA isolation.

### Fluorescein angiography

Fundus fluorescein angiography images were acquired using Μicron IV Retinal Imaging Microscope (Phoenix Technology Group, Lakewood, CO, USA) as described previously [13, 16, 17]. Images were obtained at 3–5 min (early phase) and 7–10 min (late phase) after i.p. injection of 2% fluorescein sodium (Akorn, Inc., Lake Forest, IL, USA, H12099-0711). Lesion severity was graded as follows: lesions with patchy or faint fluorescence without any leakage were assigned a score of 0 (no leakage); lesions with hyperfluorescence without changes in intensity or size were assigned a score of 1 (mild leakage); lesions with hyperfluorescence of a consistent size but increasing intensity were assigned a score of 2A (moderate leakage); and lesions with hyperfluorescence of both increasing intensity and size were assigned a score of 2B (significant leakage). The classification was performed by two blinded examiners (JHK and YJJ), and disagreements were resolved by a third examiner (JYS).

### Choroidal flat-mounts

On day 7 after laser-induced CNV induction, the eyes of the mice were removed and fixed in a solution containing 4% paraformaldehyde at RT for 30 min. The choroid and sclera were separated from the retinas to create a choroidal flat mount. To stain the eyecups, a solution of isolectin B4 conjugated to Alexa Fluor 488 (Invitrogen, Waltham, MA, USA, I21411) was incubated at 4 °C overnight. The eyecup was then mounted flat using PermaFluor aqueous mount (Thermo Fisher Scientific, Waltham, MA, USA). The flat-mounted eyecup was imaged using LSM 800 Airyscan confocal microscope (Carl Zeiss, Jena, Germany). The size of the CNV lesions was measured using ImageJ software as described in previous studies [13, 16, 17].

### Immunohistochemical staining

Mouse eyes were enucleated and fixed in a solution containing 4% paraformaldehyde at RT for 1 h followed by the incubation in 30% sucrose solution at 4 °C overnight and then embedded in an OCT compound. Cryosections of 15 µm-thickness were incubated with a blocking buffer at RT for 1 h. After incubation with primary antibodies at 4 °C overnight, the sections were incubated with secondary antibodies at RT for 1 h. Antibody information is described in Table S1.

### Cell culture

Primary human RPE (hRPE) cells (Lonza, Walkersville, MD, USA, 00194987) were used between 5 and 6 passages. The cells were cultured in basal media with added supplements of RtEGM BulletKit (Lonza, 00195409) at 37 °C in a 5% CO_2_ humidified incubator, according to the manufacturer’s instructions. The cells were seeded with 2% FBS/RtEGM media overnight and incubated with serum-free RtEGM media for 24 h. Confluent cells were treated in serum-free RtEGM containing 5 ng/mL of inflammatory cytokine mixture (ICM) including IL-1β, TNF-α, and IFN-γ from R&D system for 24 h mimicking the inflammatory state of AMD [18]. For the cell viability assay, the Cell Counting Kit 8 (Abcam, Waltham, MA, USA, ab228554) was used according to the manufacturer’s instructions.

### siRNA transfection

Predesigned siRNAs targeting human *PDK1*-4 and si*Control* (SN-1003) (Bioneer, Daejeon, Korea) were described in Table S2. siRNA transfections were performed with Lipofectamine RNAiMAX and Opti-MEM according to the manufacturer’s instructions (Thermo Fisher Scientific). In brief, the cells were cultured in a 12-well plate and transfected at 60–70% confluency with siRNA at 500 pM. The next day, the cells were incubated with the media containing ICM for 24 h.

### Quantitative real-time polymerase chain reaction (qPCR)

Total RNA was extracted from the tissues or cells using QIAzol lysis reagent (Qiagen, Hilden, Germany) and used for cDNA synthesis using a kit (Thermo Fisher Scientific). The qPCR analysis was performed with Luna Universal qPCR Master Mix (New England Biolabs, Ipswich, MA, USA) using a ViiA7 real-time PCR system (Applied Biosystems, Carlsbad, CA, USA). Specific primers used for real-time PCR are described in Table S3.

### Western blot analysis

Mouse tissues and the cells were lysed with RIPA buffer (Thermo Fisher Scientific) containing a phosphatase inhibitor cocktail (Sigma-Aldrich). The BCA protein assay (Thermo Fisher Scientific) measured protein concentrations. Nupage 4–12% Bis-Tris Mini Protein Gels (Thermo Fisher Scientific) separated the lysates. Antibody information is described in Table S1.

### Enzyme-linked immunosorbent assay (ELISA) for cytokines

After diluting 1:10 to 200 in cell culture medium, the secreted amounts of human IL6 (Thermo Fisher Scientific, 88-7066-76), IL8 (R&D system, Minneapolis, MN, USA, D8000C), and MCP1 (Thermofisher Scientific, 88-7399-76) were measured using ELISA kits, according to the manufacturer’s instructions.

### Measurement of oxygen consumption rate (OCR) and extracellular acidification rate (ECAR)

OCR was measured using an XFe96 Extracellular Flux Analyzer (Seahorse Biosciences, Inc., Billerica, MA, USA). The cells were seeded in 6-well plates at a density of 1.4 × 10^5^ cells/well. On day 3, the cells were washed and treated with ICM diluted in serum-free media with DCA or GM10395 for 24 h. Next, cells were transferred into the Xfe96 cell culture plate (Agilent Technologies, Santa Clara, CA, USA, 103794-100) with assay medium at a density of 1.0 × 10^4^ cells/well, and the plate was incubated in 5% CO_2_ at 37 °C for 5 h. The OCR assay medium consisted of XF DMEM medium pH 7.4 (Agilent Technologies, 102353) supplemented with 25 mM Glucose Solution (Sigma-Aldrich, G7528), 1 mM Pyruvate Solution (Sigma-Aldrich, S8636), and 2 mM Glutamine Solution (Thermo Fisher Scientific, 35050061). The ECAR assay medium consisted of XF DMEM medium pH 7.4 supplemented with 2 mM Glutamine Solution. Uncouplers and inhibitors were used at the indicated concentrations: oligomycin A (1 μM, Sigma-Aldrich, 75351), FCCP (carbonyl cyanide 4-(trifluoro-methoxy)phenylhydrazone, 1 μM, Sigma-Aldrich, C2920), rotenone (1 μM, Sigma-Aldrich, R8875), 2-DG (50 mM, Sigma-Aldrich, D6134) and Glucose (10 mM, Sigma-Aldrich, G7528). The nuclei were stained with DAPI solution (1 μg/mL, Thermo Fisher Scientific, 62248) and counted automatically using an ImageXpress Micro Confocal Microscope (Molecular Devices, San Jose, CA, USA) to normalize by cell number.

### Cellular superoxide detection

A mitochondrial Superoxide Indicator (MitoSOX™, Thermo Fisher Scientific) was used to detect mitochondrial superoxide formation in the cells following ICM treatment for 24 h with drugs. The cells were seeded at a density of 2 × 10^4^ cells/well in a black wall/clear bottom 96-well plate (Greiner Bio-One, Kremsmünster, Austria). At the end of the experiment, the cells were incubated for 10 min at 37 °C in PBS containing MitoSOX (5 μM) and NucBlue™ Live ReadyProbes™ Reagent solution (Thermo Fisher Scientific). Red-stained mitochondrial superoxide was image-captured using an ImageXpress Micro Confocal System. The mean fluorescence intensity from DAPI and TRITC in image planes was imaged and quantified automatically.

### Immunofluorescence for mitochondrial morphology

Cells were seeded at a density of 6 × 10^3^ cells/well in a black wall/clear bottom 96-well plate (Greiner Bio-One) and were treated with 4% paraformaldehyde for fixation, followed by permeabilization. The cells were then washed with PBS and incubated with primary antibody against TOM20 (Santa Cruz Biotechnology, Santa Cruz, CA, USA, sc-11415) at 4 °C overnight, followed by the incubation with Alexa Fluor 594-conjugated donkey anti-rabbit antibody (Thermo Fisher Scientific, A21207) at RT for 2 h. Nuclei were stained with DAPI solution (Thermo Fisher Scientific). Images were obtained from a 2.4 mm z-stack as described previously [13]. The sum of fluorescence pixels was calculated for each cell within the images. The total fluorescence intensity from each image was divided by the number of cells in that image to obtain the average fluorescence intensity per cell, as described previously [19].

### Statistics

All results were expressed as mean ± SEM. Datasets were analyzed using a two-sample *t*-test and one-way or two-way ANOVA followed by Tukey’s multiple comparison test. Data analyses were performed using Prism v. 6.0 (GraphPad Software, San Diego, CA, USA), and *P* < 0.05 was considered statistically significant.

## Results

### PDK4 activity is upregulated in the RPE of laser-induced CNV mice

**Fig. 1:**
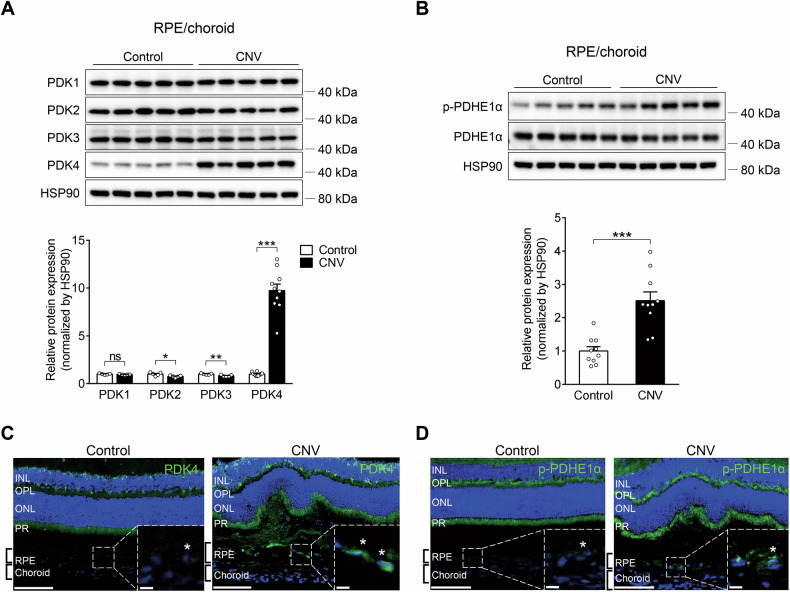
Pyruvate dehydrogenase kinase isoform 4 (PDK4) activity is upregulated in the retinal pigment epithelium (RPE) of laser-induced choroidal neovascularization (CNV) mice. **A**, **B** Protein was isolated from the RPE/choroid of control and laser-induced CNV mice, and protein lysates were subjected to immunoblotting for PDK1–4 and phosphorylated pyruvate dehydrogenase E1-alpha subunit (p-PDHE1α). Only PDK4 was upregulated, and p-PDHE1α was increased in the RPE of CNV mice relative to the control. **C**, **D** Representative confocal images of retina and choroid from control and day 1 post-CNV induction animals. PDK4^+^ cells (green) and p-PDHE1α^+^ cells (green) were present, especially in the RPE of CNV mice at higher magnifications, indicated by asterisks. Scale bar: 100 μm. Inset scale bar: 10 μm. Data are represented as mean ± SEM. *P < 0.05; **P < 0.01; ***P < 0.001 versus control (*n* = 5 mice/group). Two-tailed unpaired *t*-test. HSP90 heat shock protein 90, PDHE1α pyruvate dehydrogenase E1-alpha subunit, INL inner nuclear layer, OPL outer plexiform layer, ONL outer nuclear layer, PR photoreceptor.

To identify the specific mitochondrial PDK isoform involved in the development of neovascular AMD, we evaluated the expression of four PDK isoforms that suppress OXPHOS by PDK-mediated phosphorylation of PDHE1α (p-PDHE1α) in the laser-induced CNV mouse model. Total protein levels of PDK4 and p-PDHE1α significantly increased by 9.7 ± 0.7-fold and 2.5 ± 0.3-fold, respectively, in the RPE/choroid 1 day following CNV induction, while the levels of the other isoforms (PDK1–PDK3) remained unchanged (Fig. 1A, B). Similarly, immunofluorescence for PDK4 and p-PDHE1α identified enhanced staining in the RPE/choroid, particularly following CNV induction (Fig. 1C, D). These findings are consistent with a prior study demonstrating increased PDK4-mediated PDHE1α phosphorylation in the bowel tissues of inflammatory bowel disease patients, which was recapitulated in a mouse model [14]. By contrast, the retina tissue of the CNV model did not show changes in the levels of any PDK isoform (Fig. S1). These data suggest that increased PDK4 expression in the RPE/choroid could correlate with defective mitochondrial function during the early stages of CNV.

### Pdk4 knockout mice show reduced choroidal neovascularization

**Fig. 2:**
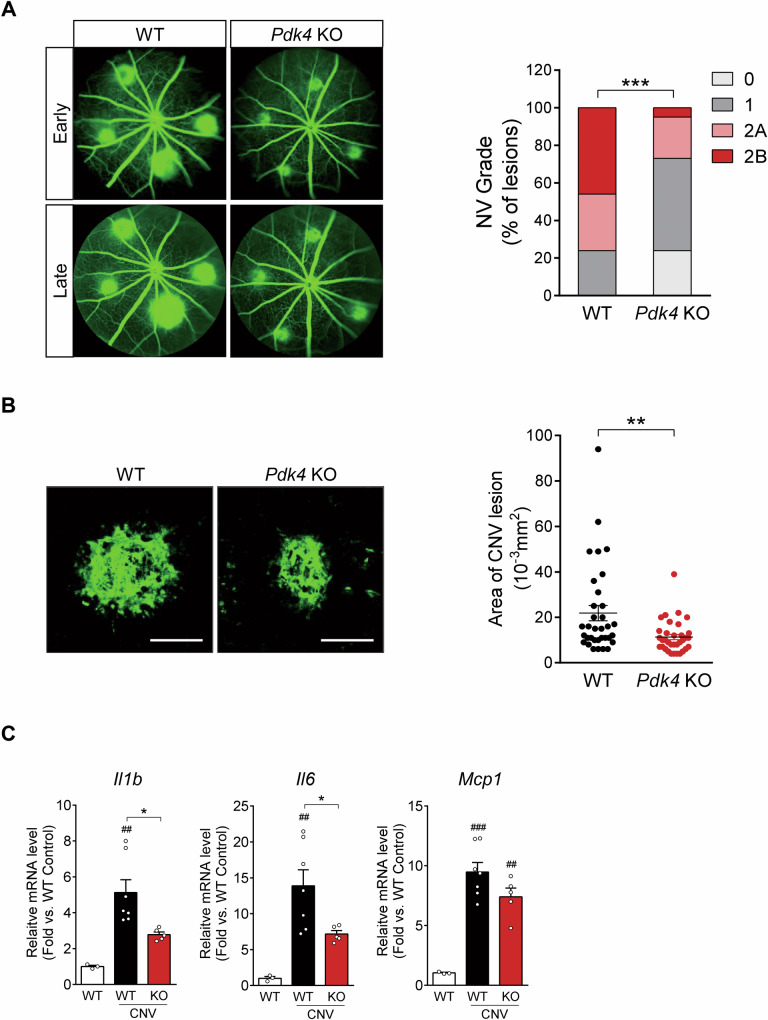
*Pdk4* knockout (*Pdk4*^−/−^) mice show reduced choroidal neovascularization (CNV). **A** Grading of CNV lesions (0, 1, 2A, and 2B) was conducted in fluorescein angiography images from wild-type (WT) and *Pdk4*^−/−^ (*Pdk4* KO) mice 7 days following CNV induction (*n* = 5 mice/group). **B** CNV lesion size was calculated in both groups. **P < 0.01; ***P < 0.001 versus wild-type CNV (*n* = 34 lesions/group). Chi-square and two-tailed unpaired *t*-test. **C** mRNA levels of proinflammatory cytokines, including *interleukin-1 beta (Il1b), interleukin-6 (Il6)*, and *monocyte chemoattractant protein-1 (Mcp1)*, were measured. Data are represented as mean ± SEM. ^##^*P* < 0.01; ^###^*P* < 0.001 versus wild type; **P < 0.01; ***P < 0.001 versus wild-type CNV (*n* = 5 mice/group). Scale bar: 100 μm. One-way ANOVA with Tukey’s multiple comparisons test. NV neovascularization.

To investigate the effect of PDK4 deficiency in CNV, we evaluated the neovascular phenotypes of wild-type *(Pdk4*^+/+^) and *Pdk4* knockout (*Pdk4*^−/−^) mice. Fluorescein angiography revealed abnormal vascular leakage from the new vessels on day 7 following CNV induction by laser photocoagulation. The proportion of grade 2B lesions with significant vascular leakage was lower in *Pdk4*^−/−^ mice than in the wild type (46% in wild-type and 5% in *Pdk4*^−/−^ mice) (Fig. 2A). Similarly, *Pdk4*^−/−^ mice exhibited a significantly smaller CNV lesion area by 48.3 ± 5.6% compared to wild-type mice (21.9 ± 3.3 in wild-type and 11.3 ± 1.2 mm^2^ × 10^−3^ in *Pdk4*^−/−^ mice) based on an examination of flat-mounted choroids stained with isolectin B4 (Fig. 2B). These findings demonstrated that PDK4 contributes to CNV, suggesting that it could be a potential therapeutic target for neovascular AMD. Inflammatory cytokines, including IL1B and MCP1, are highly expressed in the CNV mouse model and human CNV tissues [20, 21]. Thus, we compared cytokine levels between wild-type and *Pdk4*^−/−^ mice. The mRNA levels of proinflammatory cytokines, such as *Il1b* and *Il6*, were lower in the RPE/choroid of the CNV model of *Pdk4*^−/−^ mice than those in the CNV model of wild-type mice. (Fig. 2C).

### Only PDK4 gene silencing restored mitochondrial respiration in inflammation-challenged human RPE cells

**Fig. 3:**
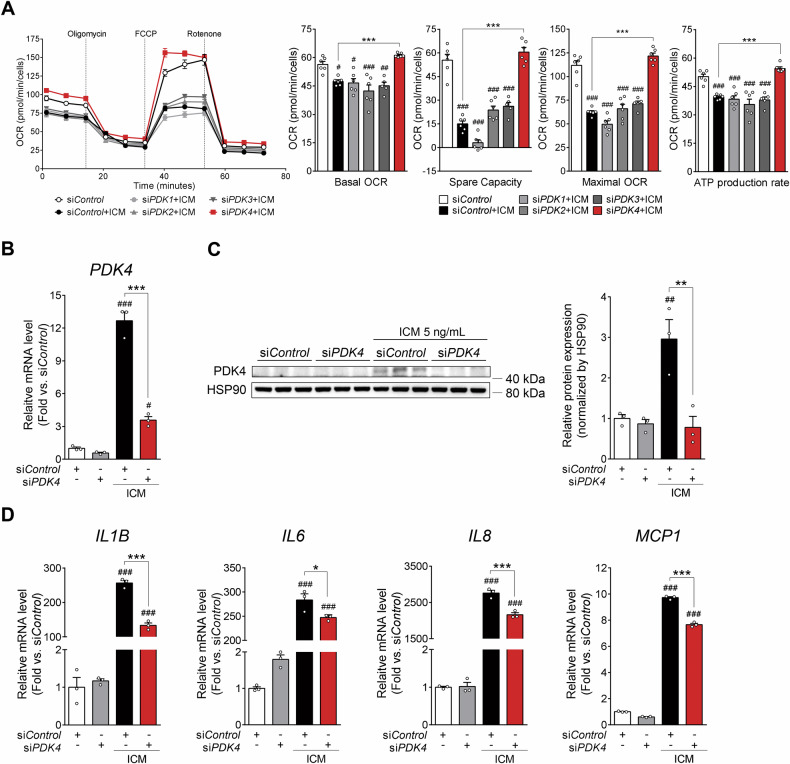
Only pyruvate dehydrogenase kinase isoform 4 (*PDK4*) gene silencing restores mitochondrial respiration in human retinal pigment epithelium (hRPE) cells under inflammatory conditions. **A** Oxygen consumption rate (OCR) in primary hRPE that were transiently transfected with si*PDK1-4* (small interfering RNA [siRNA] targeting *PDK isoform 1, 2, 3, and 4*) for 48 h followed by challenge with vehicle (PBS) or inflammatory cytokine mixture (ICM). Data are represented as mean ± SEM. ^#^*P* < 0.05; ^##^*P* < 0.01; ^###^*P* < 0.001 versus si*Control*; ***P < 0.001 versus si*Control* with ICM (*n* = 5/group). One-way ANOVA with Tukey’s multiple comparisons test. **B**, **C** *PDK4* mRNA levels and PDK4 protein levels were measured in primary hRPE cells treated with ICM following transient knockdown of *PDK4*. **D** mRNA levels of proinflammatory cytokines, including *interleukin-1 beta (IL1B), interleukin-6 (IL6), interleukin-8 (IL8), and monocyte chemoattractant protein-1 (MCP1)*, were measured in primary hRPE cells treated with ICM following transient *PDK4* knockdown using siRNA (si*PDK4*) or si*Control*. Data are expressed as mean ± SEM. ^#^*P* < 0.05; ^###^*P* < 0.001 versus si*Control*; *P < 0.05; ***P < 0.001 versus si*Control* with ICM (*n* = 3/group). Two-way ANOVA with Tukey’s multiple comparisons test.

To explore the specific roles of individual PDK isoforms on the inflammation-mediated decrease of mitochondrial respiration, we measured oxygen consumption rate (OCR) in primary hRPE cells, which were transiently transfected with either *PDK1*, *PDK2*, *PDK3*, or *PDK4* siRNA for 48 h, followed by incubation with vehicle (PBS) or ICM for an additional 24 h. Mitochondrial respiration was significantly decreased in ICM-treated human RPE cells, as evidenced by reductions in basal OCR, maximal OCR, and spare respiratory capacity. *PDK4* gene silencing but not the silencing of any other *PDK* isozyme gene strongly reversed these reductions in respiratory parameters and ATP production (Fig. 3A). The siRNA targeting inducible *PDK4* under inflammatory conditions was confirmed at both mRNA and protein levels (Fig. 3B, C). As inflammatory conditions are associated with impairment of energy metabolism of the RPE and contribute to the progression of AMD, restoring RPE oxidative metabolism could decrease inflammation. Thus, we investigated whether siRNA depletion of *PDK4* expression affects mRNA levels of *IL1B*, *IL6*, *IL8*, or *MCP1* in ICM-treated primary hRPE cells. Furthermore, it inhibited ICM-induced upregulation of *IL1B*, *IL6*, *IL8*, and *MCP1* compared with the si*Control* (Fig. 3D).

### Small-molecule PDK4 inhibitors decrease inflammatory cytokine expression

Due to the lack of commercially available PDK4 selective inhibitors, pharmacological pan-PDK inhibitors, such as dichloroacetate (DCA), which target the ATP-binding pockets of all PDK isozymes, have been used to investigate metabolic diseases associated with mitochondrial dysfunction. Our previous studies showed that PDK4 inhibition by DCA protects against obesity-associated insulin resistance via regulation of adipose tissue inflammation [22], atherosclerosis [23], and diabetes [24]. Recently, structural modifications of hit anthraquinones have resulted in a new series of allosteric PDK4 inhibitors optimally fitted to the lipoamide binding site [25]. We developed a potent allosteric PDK4 inhibitor, GM10395, and used it in the current study (Fig. S2A). ICM (5 and 10 ng/mL), DCA (1 and 10 µM), or GM10395 (0.5 and 1 µM) did not affect cell viability (Fig. S2B). We assessed the effect of DCA and GM10395 on the inflammatory response and found that DCA significantly down-regulated inflammatory cytokine expression in primary hRPE cells (Fig. S2C). These findings are consistent with those of a previous study reporting the effect of DCA on TGFβ-challenged hRPE cells [26].

### PDK4 inhibition reverses the ICM-induced metabolic switch in primary hRPE cells

**Fig. 4:**
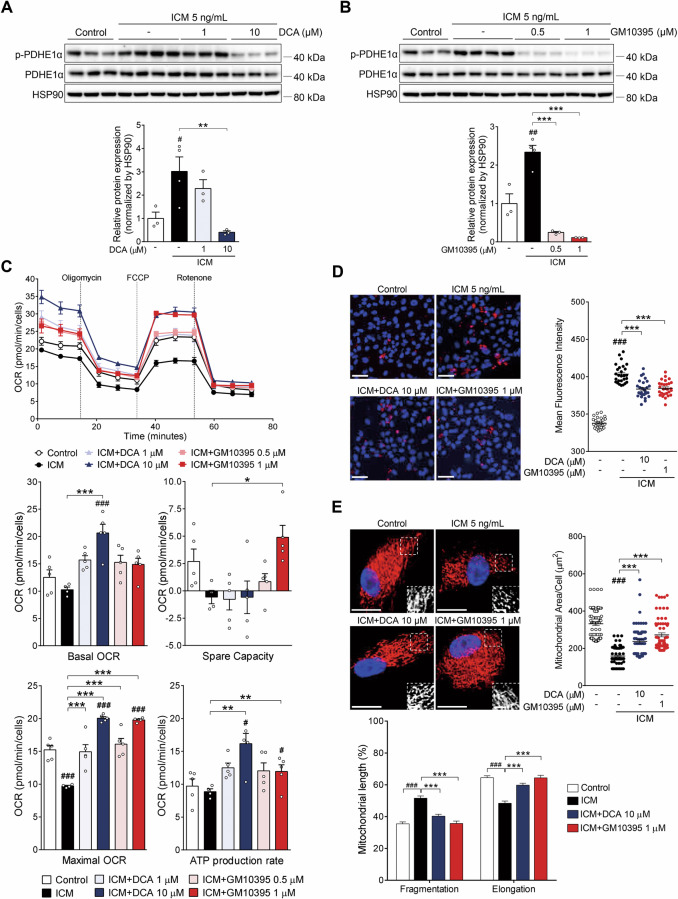
The small-molecule pyruvate dehydrogenase kinase isoform 4 (PDK4) inhibitor GM10395 promotes metabolic shift toward oxidative phosphorylation in inflammatory cytokine mixture (ICM)-treated human retinal pigment epithelium (hRPE) cells. **A**, **B** Protein was isolated from ICM-treated primary hRPE cells ± dichloroacetate (DCA; 1 and 10 μM) or GM10395 (0.5 and 1 μM) for 24 h. Lysates were subjected to immunoblotting for phosphorylated pyruvate dehydrogenase E1-alpha subunit (p-PDHE1α), and heat shock protein 90 (HSP90) ratios were determined by densitometry (*n* = 3 replicates/experiment). **C** Oxygen consumption rate (OCR) was measured in primary hRPE cells with ICM treatment for 24 h on day 3 ± DCA (1 and 10 μM) or GM10395 (0.5 and 1 μM). Basal respiration, maximal respiration, spare respiratory capacity, and ATP production rate were calculated based on the OCR response to specific inhibitors (*n* = 5/group). **D** Mitochondrial superoxide (MitoSOX) levels were measured in primary hRPE cells with ICM treatment for 24 h ± DCA (10 μM) or GM10395 (1 μM). Scale bar: 50 μm. Quantification of MitoSOX intensity (*n* = 27 microscopic fields with > 4 × 10^4^ cells counted from three independent wells). **E** Primary hRPE cells treated with ICM ± DCA (10 μM) or GM10395 (1 μM) for 24 h were fixed and stained with translocase of outer membrane 20 (TOM20) antibody to visualize mitochondrial membranes. Scale bar: 20 μm. Inset scale bar: 5 μm. Quantification of mitochondrial area, fragmentation, and elongation of > 65 cells counted for each condition. Data are represented as mean ± SEM. ^#^*P* < 0.05; ^##^*P* < 0.01; ^###^*P* < 0.001 versus control; *P < 0.05; **P < 0.01; ***P < 0.001 versus ICM group. One-way ANOVA with Tukey’s multiple comparisons test.

Next, we determined if DCA or GM10395 affected the level of p-PDHE1α in ICM-treated hRPE cells (Fig. 4A, B). GM10395 at 0.5 µM was as effective in decreasing the level of p-PDHE1α as DCA, a pan-PDK inhibitor, at 10 µM. We previously reported that inflammation-induced metabolic stress of the RPE and decreased mitochondrial respiration exacerbate CNV [13]. To determine whether PDK regulates metabolic reprogramming in this context, we evaluated the effect of DCA and GM10395 on OCR and ECAR in ICM-treated primary hRPE cells. The maximal OCR, which was decreased by 36.4 ± 0.7% in ICM-treated cells, was significantly restored by 10 µM DCA or 1 µM GM10395, which was similar to the increase in the ATP production rate by PDK4 inhibitors (Fig. 4C). Glycolysis, which was increased by 61.1 ± 0.7% in ICM-treated primary hRPE cells, was significantly decreased by 10 µM DCA and 0.5 and 1 µM GM10395 (Fig. S3).

Next, we evaluated the effect of DCA and GM10395 on mitochondrial superoxide levels using MitoSOX staining. The ICM-treated primary hRPE cells exhibited a 19.5 ± 0.7% increase in superoxide levels, which was significantly reduced by DCA or GM10395 treatment (Fig. 4D). Immunostaining of mitochondria with Tom20 showed that the mitochondrial area, which was reduced by 52.1 ± 1.6% in ICM-treated primary hRPE cells, was significantly restored by DCA (71.5 ± 2.8%) and GM10395 (82.2 ± 3.2%) (Fig. 4E). Similarly, the increased mitochondrial fragmentation in ICM-treated hRPE cells was significantly reversed by DCA and GM10395 (Fig. 4E).

To assess the anti-inflammatory effect of GM10395, we measured mRNA and protein levels of proinflammatory cytokines after DCA or GM10395 treatment of ICM-treated primary hRPE cells. Inhibition of PDK4 by DCA or GM10395 significantly attenuated the upregulation of inflammatory cytokines (Fig. S4). Previous studies have shown that mixed cytokine treatment in hRPE cells upregulated NFkB expression and increased cytokine secretion, mimicking the inflammatory CNV condition [18, 27, 28]. Furthermore, Kutty et al. showed the anti-inflammatory effect of an antioxidant occurs via regulation of the NFkB pathway in ICM-treated hRPE cells [29]. Our previous study showed that PDH activation by reversing the metabolic reprogramming of RPE cells under inflammatory conditions significantly decreased cytokine secretion via the regulation of NFkB pathway [13]. These findings demonstrate that small-molecule inhibition of PDK4 in RPE cells increases mitochondrial respiration, resulting in reduced cytokine levels under inflammatory conditions.

### GM10395-mediated PDK4 inhibition alleviates laser-induced CNV

**Fig. 5:**
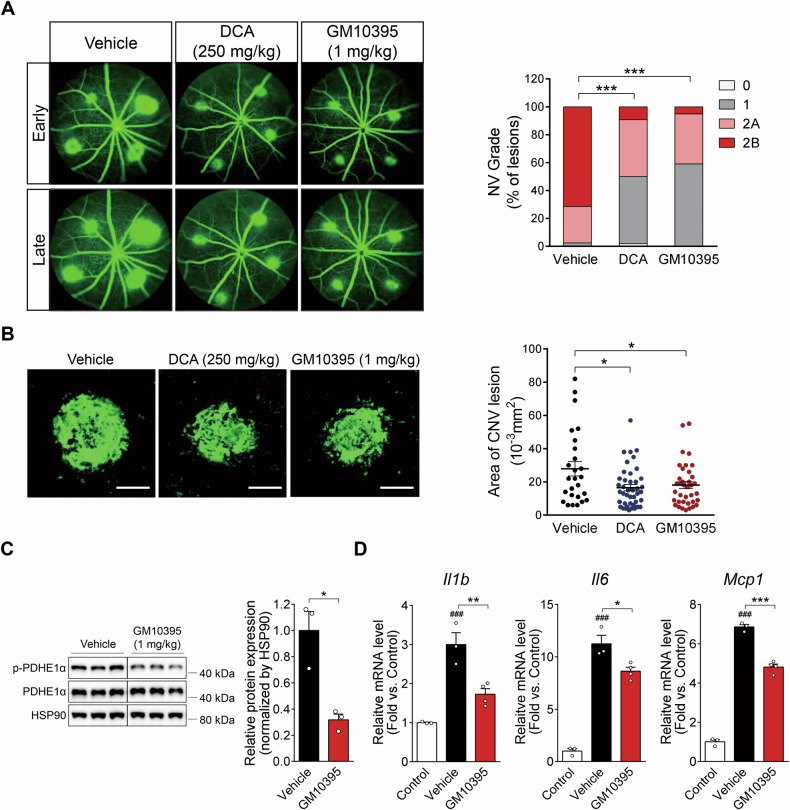
The pyruvate dehydrogenase kinase isoform 4 (PDK4) inhibitor GM10395 suppresses proinflammatory cytokines and alleviates laser-induced choroidal neovascularization (CNV). **A** Grading of CNV lesions was conducted in fluorescein angiography images from the vehicle, dichloroacetate (DCA; 250 mg/kg), and GM10395 (1 mg/kg)-treated CNV mice 7 days following CNV induction (*n* = 5 mice/group). ***P < 0.001 versus vehicle, Chi-square. **B** CNV lesion size was calculated using choroidal flat-mounts. (*n* = 34 lesions/group). Scale bar: 100 μm. Data are expressed as mean ± SEM. *P < 0.05 versus vehicle. One-way ANOVA with Tukey’s multiple comparisons test. **C** Protein was isolated from retinal pigment epithelium (RPE)/choroid of the vehicle and GM10395 (1 mg/kg)-treated CNV mice 1 day after CNV induction. Lysates were subjected to immunoblotting for phosphorylated pyruvate dehydrogenase E1-alpha subunit (p-PDHE1α). The lanes were run on the same gel but were noncontiguous. Data are expressed as mean ± SEM. *P < 0.05 versus vehicle (*n* = 3/group). Two-tailed unpaired *t*-test. **D** mRNA levels of the proinflammatory cytokines interleukin-1 beta (*Il1b*), interleukin-6 (*Il6*), and monocyte chemoattractant protein-1 (*Mcp1*) were measured in the RPE/choroid of control mice, and vehicle- and GM10395 (1 mg/kg)-treated CNV mice 3 days after CNV induction. (*n* = 3/group). Data are expressed as mean ± SEM. ^###^*P* < 0.001 versus control; *P < 0.05; **P < 0.01; ***P < 0.001 versus vehicle. One-way ANOVA with Tukey’s multiple comparisons test. HSP90, heat shock protein 90; NV, neovascularization; PDHE1α, pyruvate dehydrogenase E1-alpha subunit.

To evaluate the anti-angiogenic efficacy of DCA and GM10395, we orally administered the CNV model mice with 250 mg/kg DCA or 1 mg/kg GM10395. Both DCA and GM10395 significantly decreased the proportion of clinically significant Grade 2B CNV lesions compared with vehicle on day 7 following CNV induction (Fig. 5A). Likewise, CNV size was 40.9 ± 6.7% lower in the DCA group and 35.4 ± 7.6% lower in the GM10395 group than in the vehicle group, as assessed using choroidal flat-mount preparations (Fig. 5B). GM10395 decreased the p-PDHE1α levels in the RPE of CNV mice by 68.3 ± 4.5% compared to vehicle (Fig. 5C), which is similar to the decrease in the p-PDHE1α levels by GM10395 in primary hRPE cells (Fig. 4B). The above data indicate that oral administration of the PDK4 specific inhibitor GM10395 protected from inflammatory and metabolic reprogramming of RPE in the CNV model. Consistent with the in vitro data showing reduced proinflammatory cytokine levels (Fig. S4), mRNA levels of proinflammatory cytokines, such as *Il1b*, *Il6*, and *Mcp1*, were also decreased in the RPE/choroid of CNV animals (Fig. 5D). Taken together, these findings demonstrate that PDK4 inhibition alleviates CNV and has anti-inflammatory effects.

## Discussion

The RPE relies almost exclusively on mitochondria OXPHOS for ATP production, which allows photoreceptors to utilize glucose transported from the choroid through the RPE [30]. This process is highly regulated by suppressing RPE glycolysis to preserve glucose for photoreceptors. However, AMD disrupts this metabolic ecosystem, causing the RPE to rely on glycolysis to fulfill its energetic needs during metabolic reprogramming [30].

Previous studies evaluated the link between PDK-mediated glycolytic metabolic shift and upregulation of proinflammatory cytokines in neural tissues such as dorsal root ganglion, which causes painful diabetic neuropathy [31]. Furthermore, pharmacologic inhibition of pan-PDK with DCA and its genetic deletion attenuates proinflammatory cytokine expression in this context, alleviating neuroinflammation in the spinal cord. Similarly, the metabolic shift in RPE mitochondria promotes a proinflammatory environment, exacerbating the neovascular phenotypes of AMD. However, the link between metabolic reprogramming in the human RPE and the neovascular phenotypes of CNV remains incompletely understood. Thus, in the present study, we evaluated the functional effects of PDK4-mediated RPE glycolytic reprogramming using genetic ablation and small-molecule targeting specific PDK4, identifying PDK4 as a modulator of mitochondrial dynamics in this context.

Among PDK isoforms, only PDK4 was upregulated in the RPE of CNV animals. Furthermore, *Pdk4* ablation alleviated CNV. In vitro studies with primary hRPE cells demonstrated that only *Pdk4* silencing restored ICM-induced downregulation of OCR and upregulation of proinflammatory cytokines. Thus, we proposed PDK4 as a potential therapeutic target for CNV.

DCA, a structural analog of pyruvate and an inhibitor for PDKs [32], has protective effects in various neurological disorders including neurodegenerative diseases [33]. However, DCA has severe systemic side effects, including peripheral neuropathy [34, 35], necessitating the development of alternative PDK inhibitors, especially those targeting PDK4. Recently, we developed a new small-molecule PDK4 inhibitor, GM10395, and it showed a protective effect on mitochondrial dysfunction in ischemia-reperfusion kidney injury mouse models [36].

In the current study, GM10395 significantly increased maximal respiration and spare respiration. Likewise, it significantly decreased the glycolysis of hRPE cells. Furthermore, GM10395 alleviated inflammation-induced oxidative stress and mitochondrial fission. This indicates that enhancing mitochondrial activity renders the RPE more resistant to oxidative stress, further supporting the critical role of RPE mitochondria in CNV [37]. Together, our data suggest that inhibition of PDK4 protects the RPE from the inflammatory metabolic shift to aerobic glycolysis.

Inflammatory activation of the RPE changes mitochondrial dynamics and upregulates the expression of proinflammatory cytokines. A previous study reported that inhibiting LPS-induced metabolic reprogramming reduced releasing proinflammatory cytokines [38]. Similarly, GM10395 dose-dependently suppressed proinflammatory cytokine production. GM10395 attenuated the inflammatory response in the RPE by increasing mitochondrial metabolism via the inhibition of PDK4 expression, alleviating neovascularization.

**Fig. 6:**
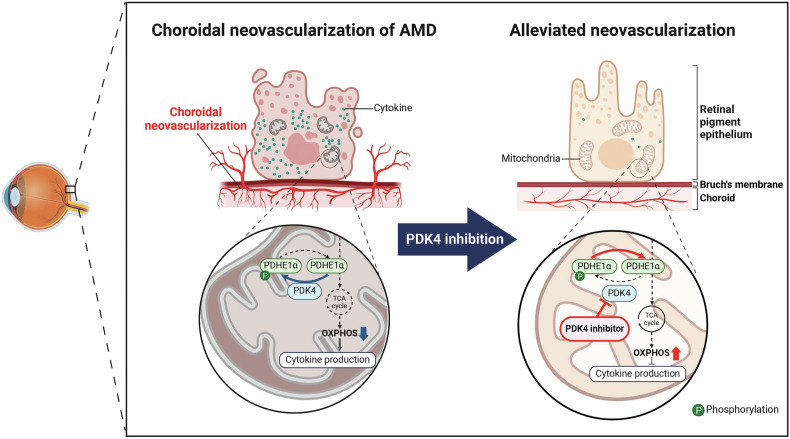
Diagram of the role of the pyruvate dehydrogenase kinase isoform 4 (PDK4) in choroidal neovascularization (CNV). PDK4 is upregulated in the retinal pigment epithelium (RPE) of laser-induced CNV mice. PDK4 inhibitors restore mitochondrial respiration and alleviate the CNV.

As shown in Fig. 6, we report that CNV is associated with the upregulation of PDK4 expression and increased levels of p-PDHE1α, causing a shift to glycolysis and inflammation. Genetic ablation of *PDK4* and a small-molecule PDK4 inhibitor alleviated CNV, which was accompanied by decreased inflammation. These findings suggest that small-molecule PDK4 inhibitors administrated orally could be utilized to develop a new class of neovascular AMD therapeutics, which might also be applicable for the therapy of other spectrums of neovascular diseases.

### Supplementary information

Supplementary Information_clean version

Full and uncropped western blots

## Data Availability

All data generated or analyzed during this study are included in the main text and the supplementary information files.
