# Supplementary material for: PDK4-mediated metabolic reprogramming is a potential therapeutic target for neovascular age-related macular degeneration
Source: Cell Death Dis. 2024 Aug 9;15(8):582. doi: 10.1038/s41419-024-06968-0 (PMC11316003; doi:10.1038/s41419-024-06968-0)
Supplement: Supplementary file 1 — Supplementary Information_clean version [file 41419_2024_6968_MOESM1_ESM.doc]

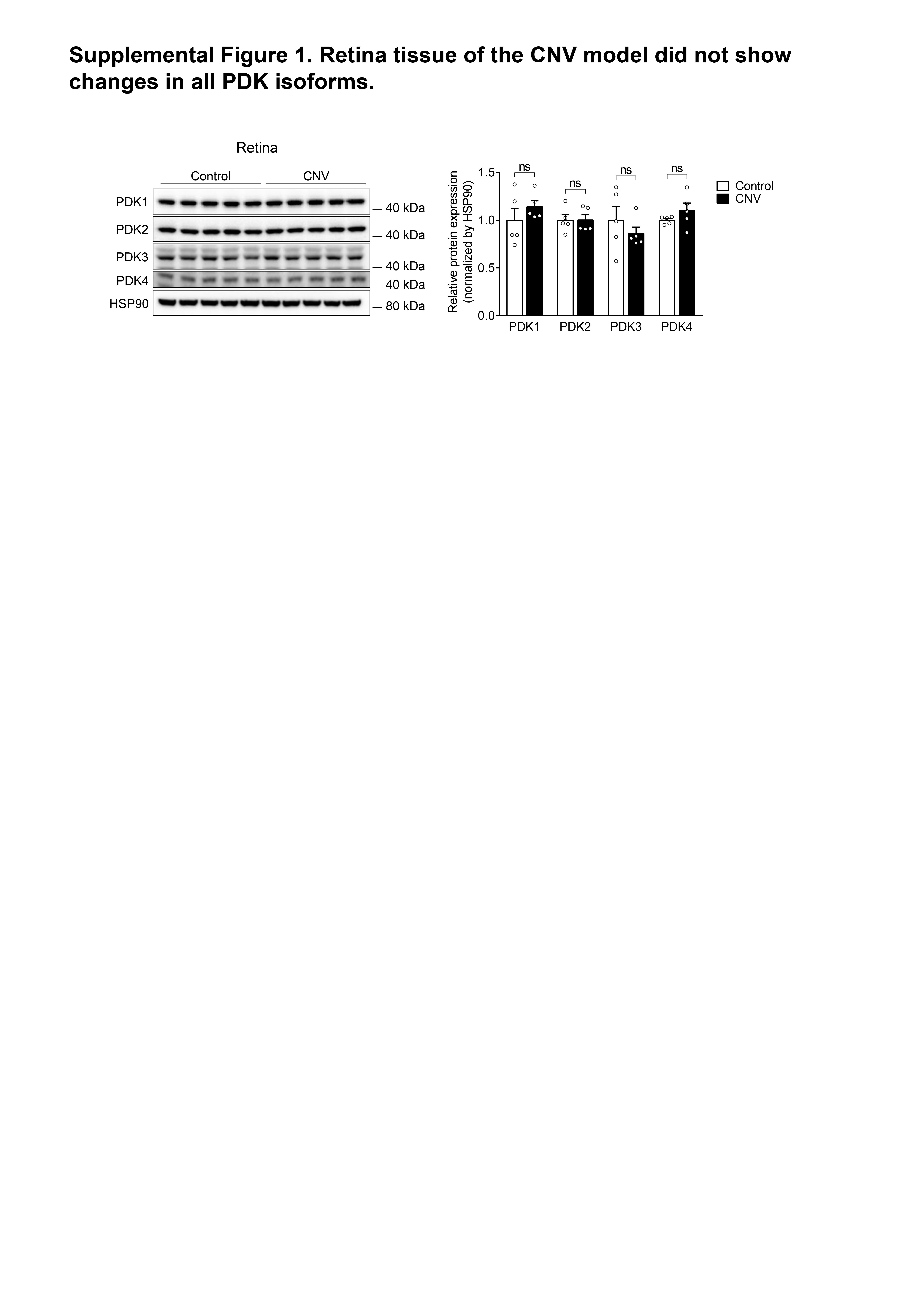


**Fig. S1 Retina tissue from nerve fiber layer to photoreceptor layer in the choroidal neovascularization (CNV) model did not show changes in expression of any pyruvate dehydrogenase kinase (PDK) isoform.** Proteins were isolated from the retina of the control and laser-induced CNV mice, and subjected to immunoblotting for PDK1, PDK2, PDK3, or PDK4. Data are represented as mean ± SEM. ns, not significant (*n* = 5 mice/ group). Two-tailed unpaired *t*-test. HSP90 indicates heat shock protein 90.


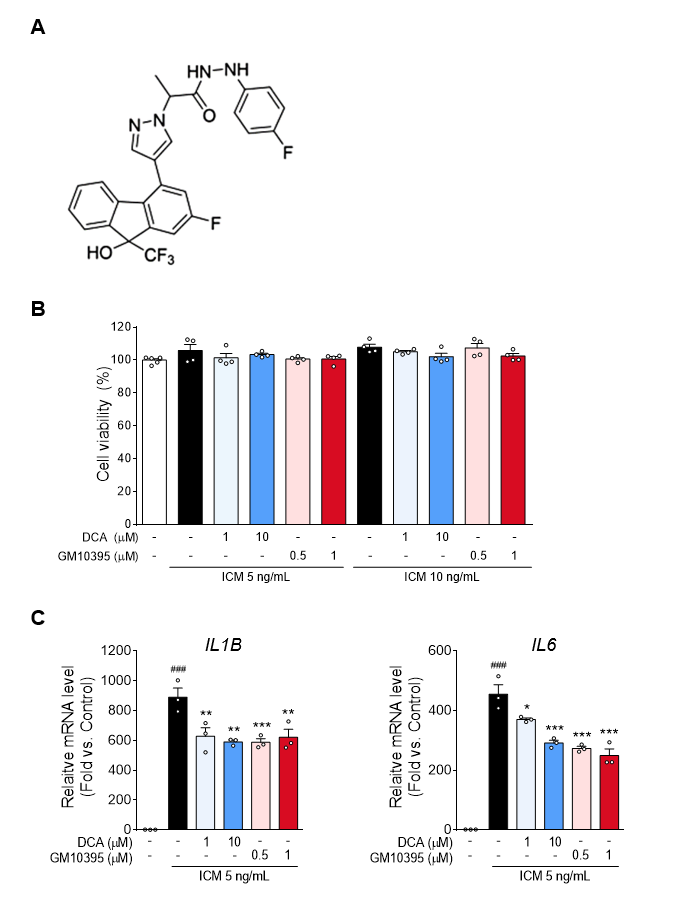


**Fig. S2 Structure of a small-molecule pyruvate dehydrogenase kinase isoform 4 inhibitor GM10395 and the effective dose range.** (**A**) Structure of the GM10395.

(**B**) Cell viability of primary human retinal pigment epithelium (hRPE) cells treated with or without ICM (5 and 10 ng/mL), DCA (1 and 10 µM), or GM10395 (0.5 and 1 µM) for 24 hours (n = 5). (**C**) mRNA levels of the proinflammatory cytokine, including interleukin-1 beta (*IL1B*) and interleukin-6 (*IL6*)*,* were measured in inflammatory cytokine mixture (ICM)-induced primary hRPE cells treated with or without sodium dichloroacetate (DCA; 1 and 10 μM) or GM10395 (0.5 and 1 μM) for 24 hours (n = 3). Data are represented as mean ± SEM. ^###^*P* < 0.001 versus Control; ^*^*P* < 0.05; ^**^*P* < 0.01; ^***^*P* < 0.001 versus ICM group. One-way ANOVA with Tukey’s multiple comparisons test.


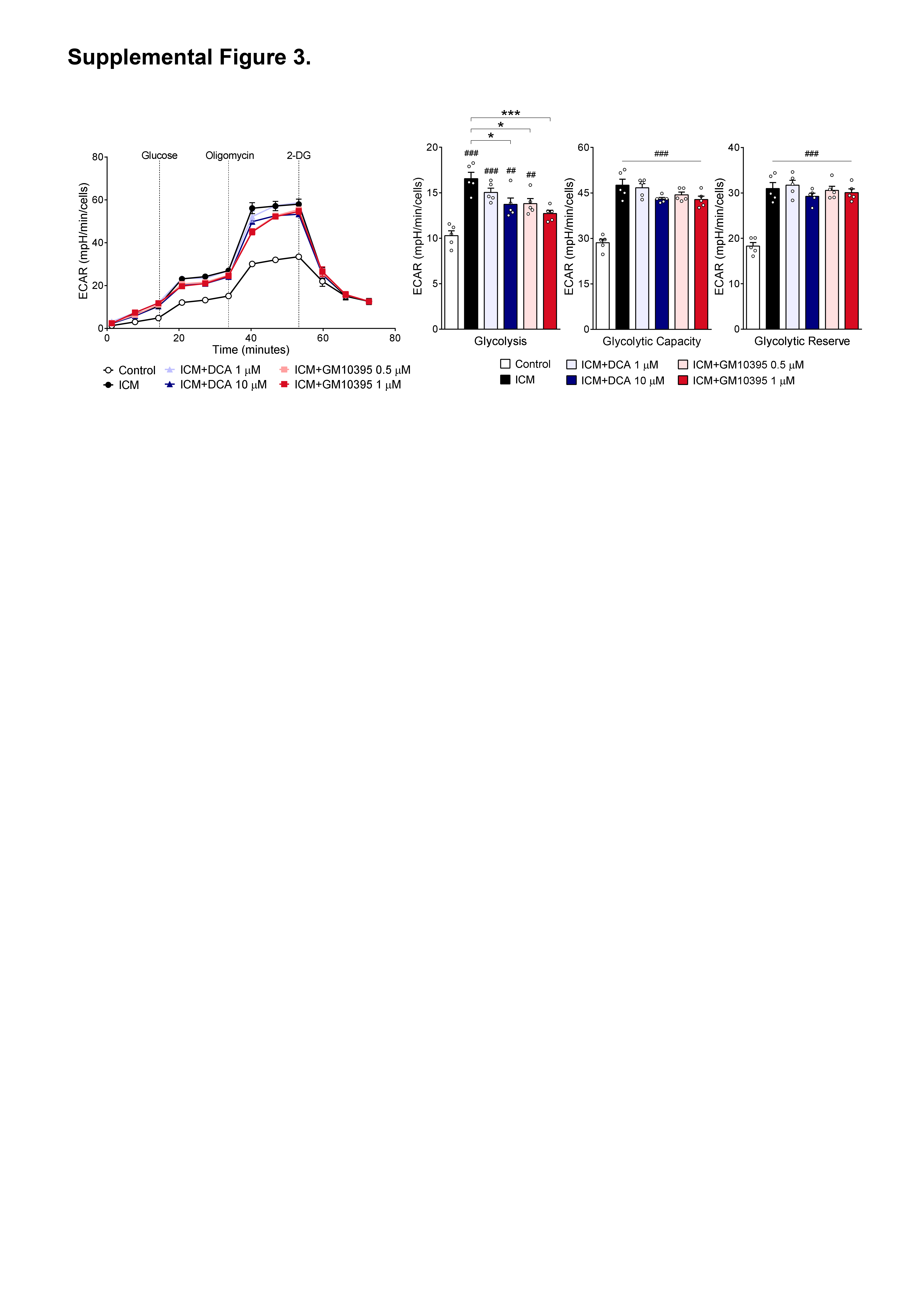


**Fig. S3 Glycolysis, which was increased in inflammatory cytokine mixture (ICM)-treated primary human retinal pigment epithelium (hRPE) cells, was significantly decreased by sodium dichloroacetate (DCA) and GM10395.** Extracellular acidification rate (ECAR) was measured in primary hRPE cells with ICM-treatment for 24 hours ± DCA (1 and 10 μM) or GM10395 (0.5 and 1 μM). Glycolysis, glycolytic capacity, and glycolytic reserve were calculated based on the ECAR response to specific inhibitors (*n* = 5/group). Data are represented as mean ± SEM. ^##^*P* < 0.01; ^###^*P* < 0.001 versus control; **P* < 0.05; ****P* < 0.001 versus ICM group. One-way ANOVA with Tukey’s multiple comparisons test.


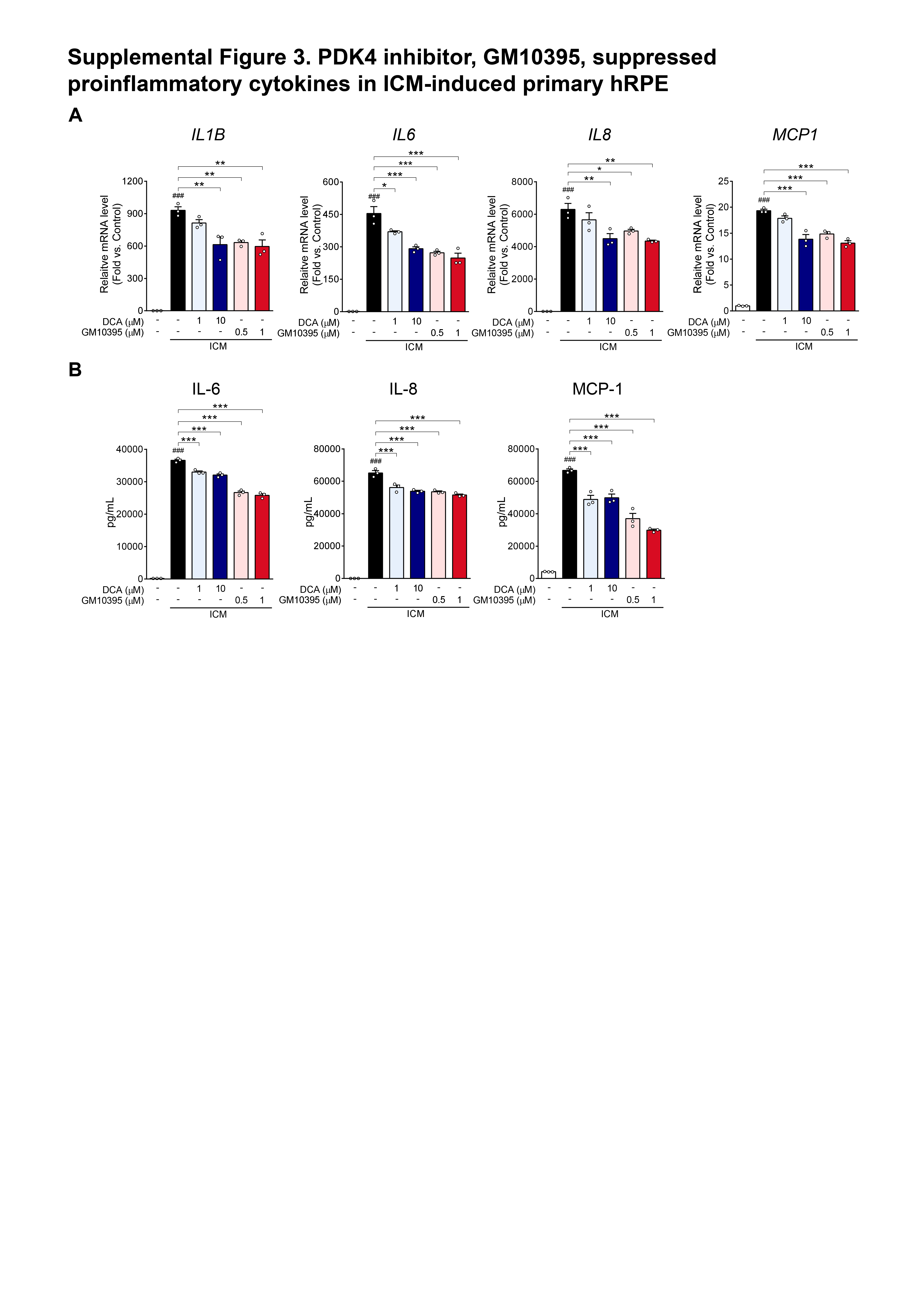


**Fig. S4 GM10395 as a pyruvate dehydrogenase kinase isoform 4 (PDK4) inhibitor suppressed the high levels of proinflammatory cytokines in inflammatory cytokine mixture (ICM)-treated primary human retinal pigment epithelium (hRPE) cells.** (**A**) mRNA levels of the proinflammatory cytokine, including interleukin-1 beta (*IL1B*), interleukin-6 (*IL6*), interleukin-8 (*IL8*), and monocyte chemoattractant protein-1 (*MCP1*), were measured in ICM-treated primary hRPE cells in the presence and absence of sodium dichloroacetate (DCA; 1 and 10 μM) or GM10395 (0.5 and 1 μM) for 24 hours (*n* = 3/group). (**B**) Protein levels of the proinflammatory cytokine, including IL-6, IL-8, and MCP-1, were measured in media collected from ICM-treated primary hRPE cells in the presence and absence of DCA (1 and 10 μM) or GM10395 (0.5 and 1 μM) for 24 hours (*n* = 3/group). Data are represented as mean ± SEM. ^###^*P* < 0.001 versus control; **P* < 0.05; ***P* < 0.01; ****P* < 0.001 versus ICM only. One-way ANOVA with Tukey’s multiple comparisons test.

## **Table S1. Primary and secondary antibodies used for immunohistochemical staining**

| **Target antigen** | **Vendor or Source** | **Catalog #** | **Working concentration** |
| --- | --- | --- | --- |
| p-PDHE1α | MilliporeSigma | AP1062 | 1/100 (IF)  1/1000 (IB) |
| PDK4 | Sigma-Aldrich | HPA056731 | 1:200 (IF) |
| Alexa Fluor 488-conjugated  donkey anti-rabbit antibody | Thermo Fisher Scientific | A21206 | 1:500 (IF) |
| Translocase of outer  mitochondrial membrane 20 | Santa Cruz Biotechnology | Sc-11415 | 1/100 (IF) |
| Alexa Fluor 594-conjugated  donkey anti-rabbit antibody | Thermo Fisher Scientific | A21207 | 1/100 (IF) |
| PDK1 | Enzo Life Sciences | ADI-KAP-PK112 | 1/1000 (IB) |
| PDK2 | Abcam | Ab68164 | 1/1000 (IB) |
| PDK3 | Abnova | PAB4563 | 1/1000 (IB) |
| PDK4 | Abcam | Ab214938 | 1/1000 (IB) |
| PDK4 | Abcam | Ab110336 | 1/1000 (IB) |
| Pyruvate dehydrogenase | Cell Signaling Technology | 2784 | 1/1000 (IB) |
| Heat shock protein 90 | Cell Signaling Technology | 4874 | 1/1000 (IB) |
| β-actin | Sigma-Aldrich | A5441 | 1/5000 (IB) |

## p-PDHE1α, phosphorylated pyruvate dehydrogenase E1-alpha subunit; PDK, pyruvate dehydrogenase kinase isoform

## **Table S2**. Human siRNA sequences used for siRNA transfection

| **Gene** | **Sense (5’-3’)** | **Antisense (5’-3’)** |
| --- | --- | --- |
| *PDK1* | GUGUAGAUUAGAGGGAUGU | ACAUCCCUCUAAUCUACAC |
| *PDK2* | GGAAAGGAGUUACACCCGU | ACGGGUGUAACUCCUUUCC |
| *PDK3* | CUAAACACAUAGGAAGUAU | AUACUUCCUAUGUGUUUAG |
| *PDK4* | CUCUUCAGUGCAAUUGGU | AACCAAUUGCACUGAAGAG |

*PDK, pyruvate dehydrogenase kinase isoform 1-4*

## **Table S3. Primer sequences used for the real-time quantitative reverse transcriptase polymerase chain reaction**

| **Species** | **Gene** | **Forward primer (5’-3’)** | **Reverse primer (5’-3’)** |
| --- | --- | --- | --- |
| Mouse | *Il1b* | AGTTGACGGACCCCAAAAGAT | GTTGATGTGCTGCTGCGAGA |
|  | *Il6* | AAGTCGGAGGCTTAATTACACATGT | CCATTGCACAACTCTTTTCTCATT |
|  | *Mcp1* | GCATCCACGTGTTGGCTCA | CTCCAGCCTACTCATTGGGATCA |
|  | *36b4* | ACCTCCTTCTTCCAGGCTTT | CTCCAGTCTTTATCAGCTGC |
| Human | *IL1B* | ATGCACCTGTACGATCACTG | ACAAAGGACATGGAGAACACC |
|  | *IL6* | AGTGAGGAACAAGCCAGAGC | CAGGGGTGGTTATTGCATCT |
|  | *IL8* | GACATACTCCAAACCTTTCCACCC | CCAGACAGAGCTCTCTTCCATCAG |
|  | *MCP1* | CTCATAGCAGCCACCTTCATTC | TCACAGCTTCTTTGGGACACTT |
|  | *PDK4* | GTATGTTCCTCCTCACCTCCATCA | TGTTGCCCGCATTGCAT |
|  | *36B4* | CAACCCAGCTCTGGAGAAAC | GTGAGGTCCTCCTTGGTGAA |

*Il1b, interleukin-1 beta; il6, interleukin-6; il8, interleukin-8; Mcp1, monocyte chemoattractant protein-1; PDK4, pyruvate dehydrogenase isoform 4*
