# Supplementary material for: PDK4-mediated metabolic reprogramming is a potential therapeutic target for neovascular age-related macular degeneration
Source: Cell Death Dis. 2024 Aug 9;15(8):582. doi: 10.1038/s41419-024-06968-0 (PMC11316003; doi:10.1038/s41419-024-06968-0)
Supplement: Supplementary file 2 — Full and uncropped western blots [file 41419_2024_6968_MOESM2_ESM.pdf]

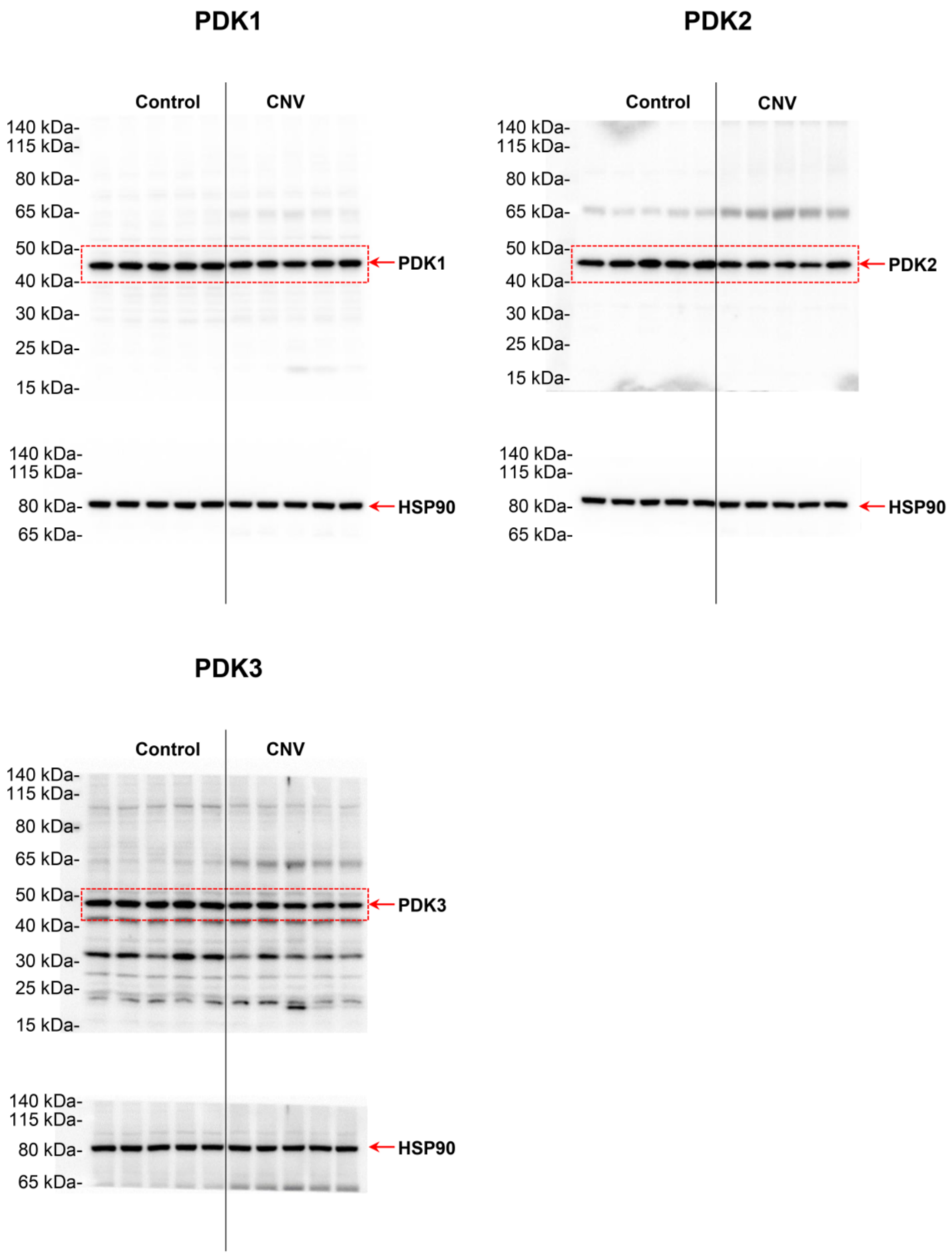

Full uncropped immunoblots of PDK1, PDK2 and PDK3 from **Fig. 1A.** The red-dotted rectangle indicates the portion of the blot displayed in the manuscript.

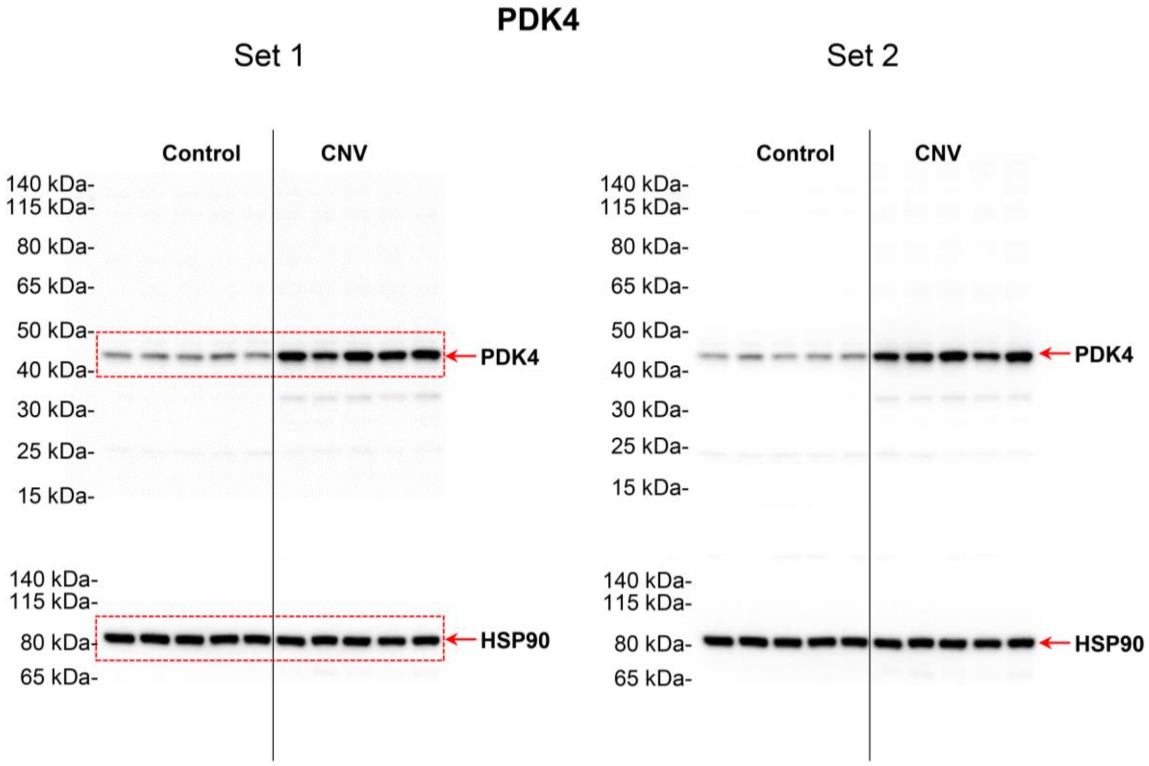

Full uncropped immunoblots of PDK4 and HSP90 from **Fig. 1A.** The red-dotted rectangle indicates the portion of the blot displayed in the manuscript.

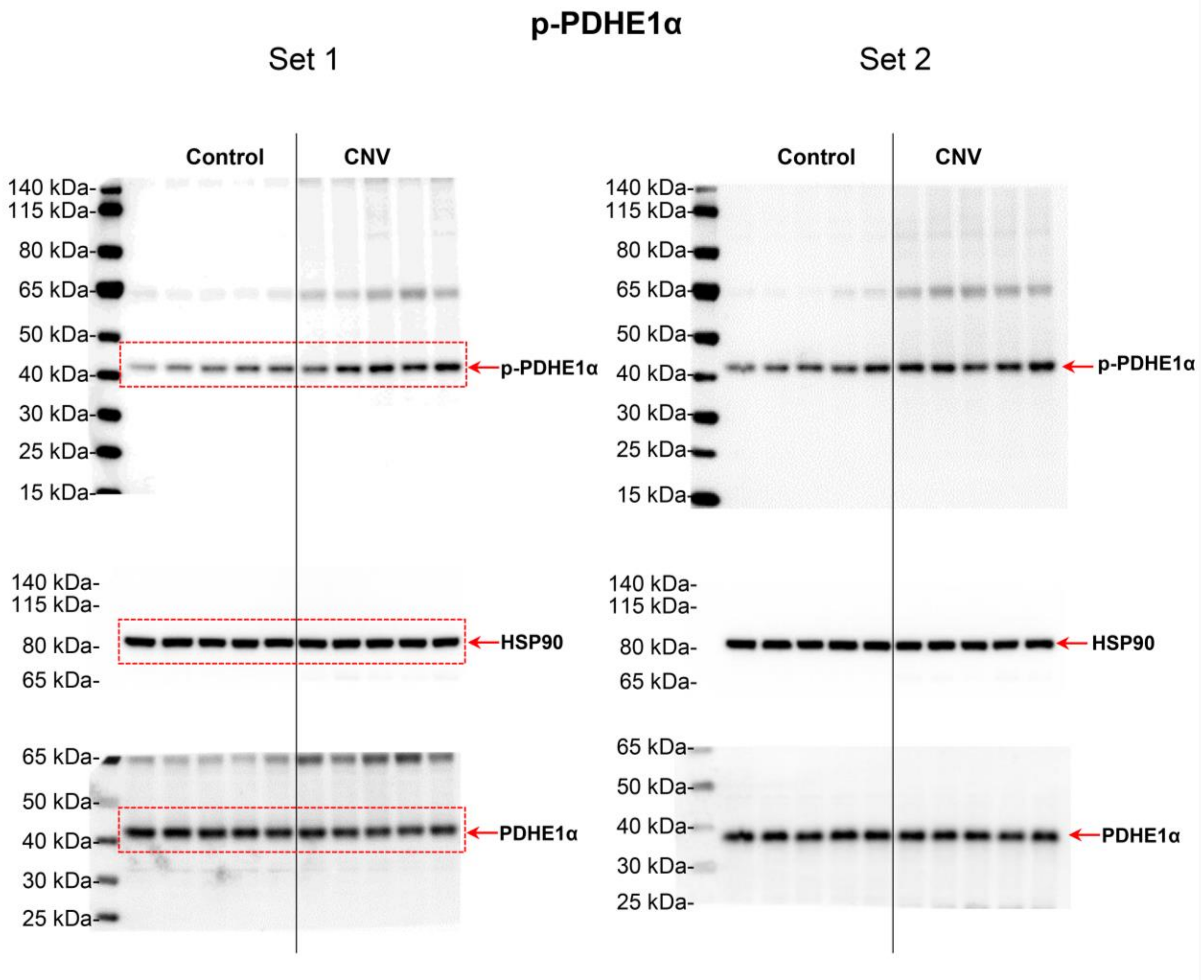

Full uncropped immunoblots of p-PDHE1α, PDHE1α and HSP90 from **Fig. 1B.** The red-dotted rectangle indicates the portion of the blot displayed in the manuscript.

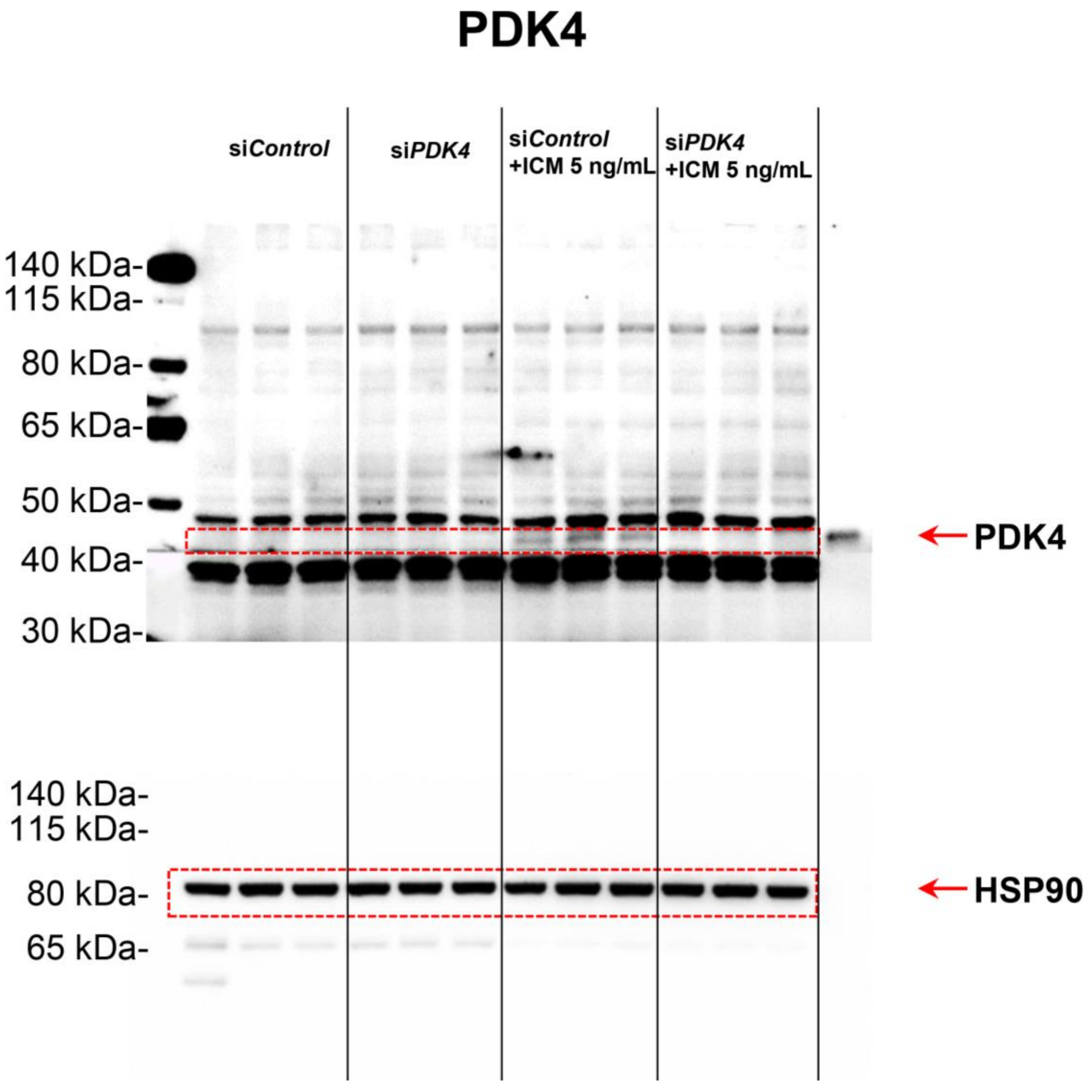

Full uncropped immunoblots of PDK4 and HSP90 from **Fig 3C.** The red-dotted rectangle indicates the portion of the blot displayed in the manuscript.

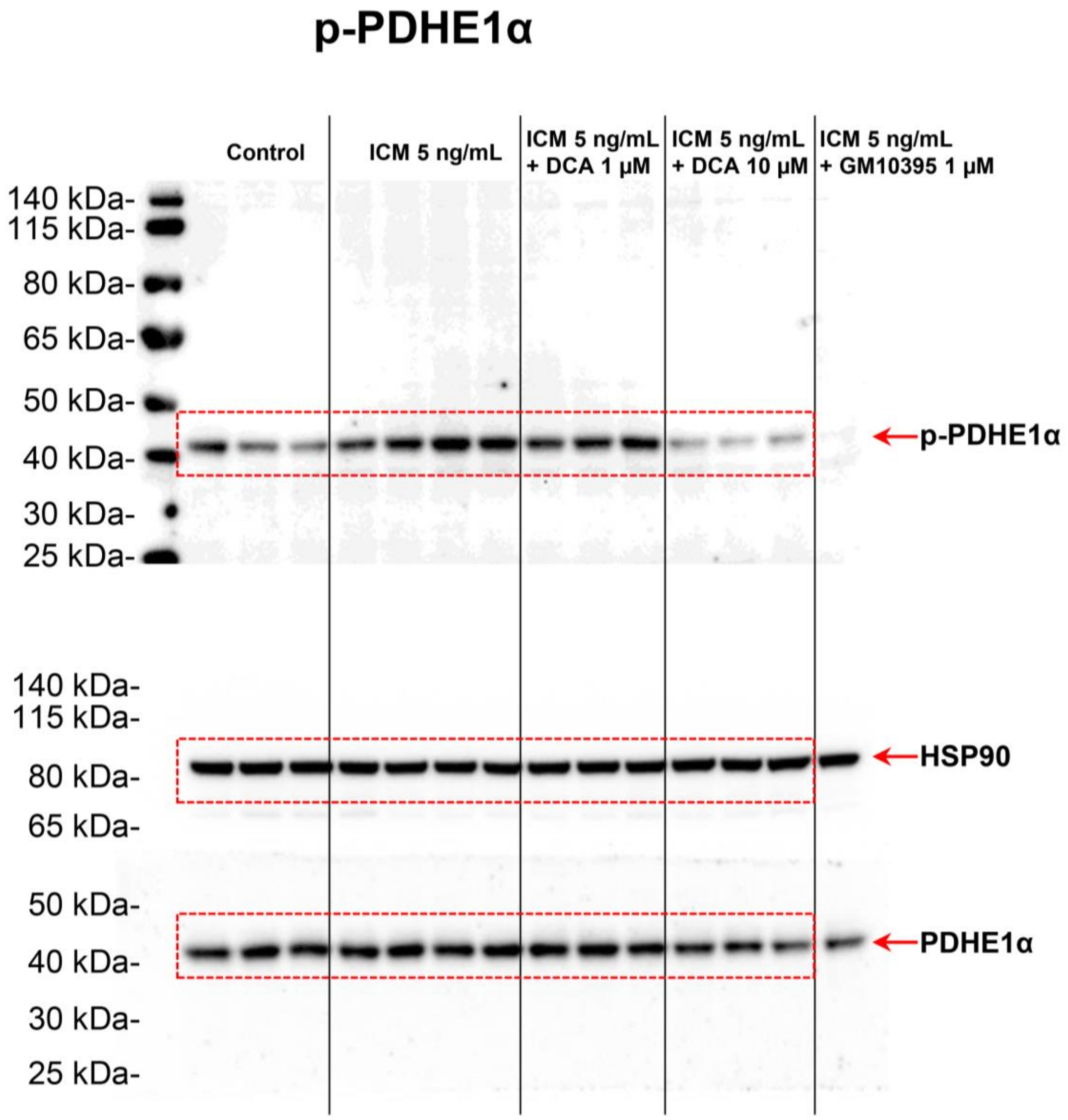

Full uncropped immunoblots of p-PDHE1α, PDHE1α and HSP90 from **Fig 4A.** The red-dotted rectangle indicates the portion of the blot displayed in the manuscript.

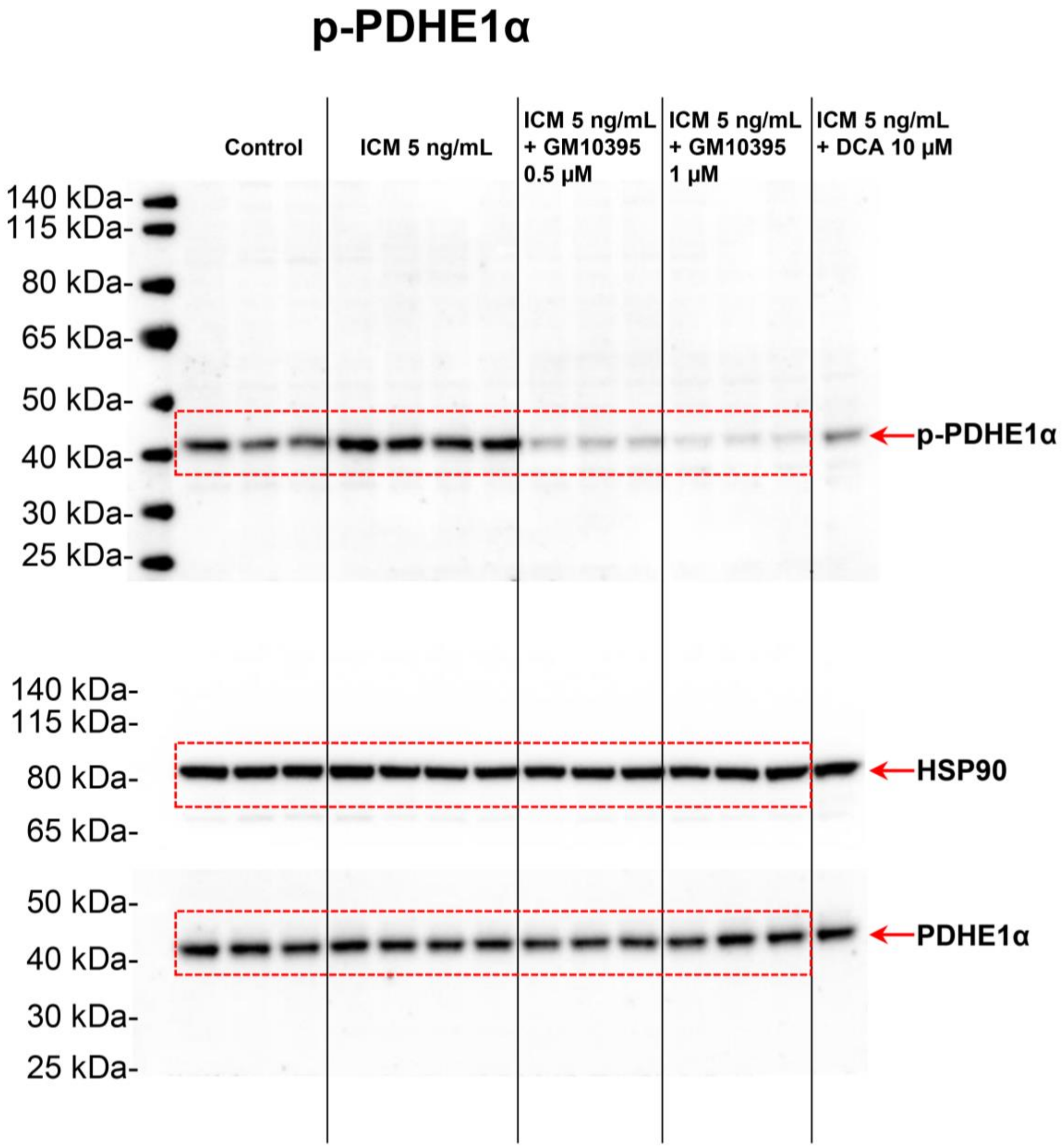

Full uncropped immunoblots of p-PDHE1α, PDHE1α and HSP90 from **Fig. 4B.** The red-dotted rectangle indicates the portion of the blot displayed in the manuscript.

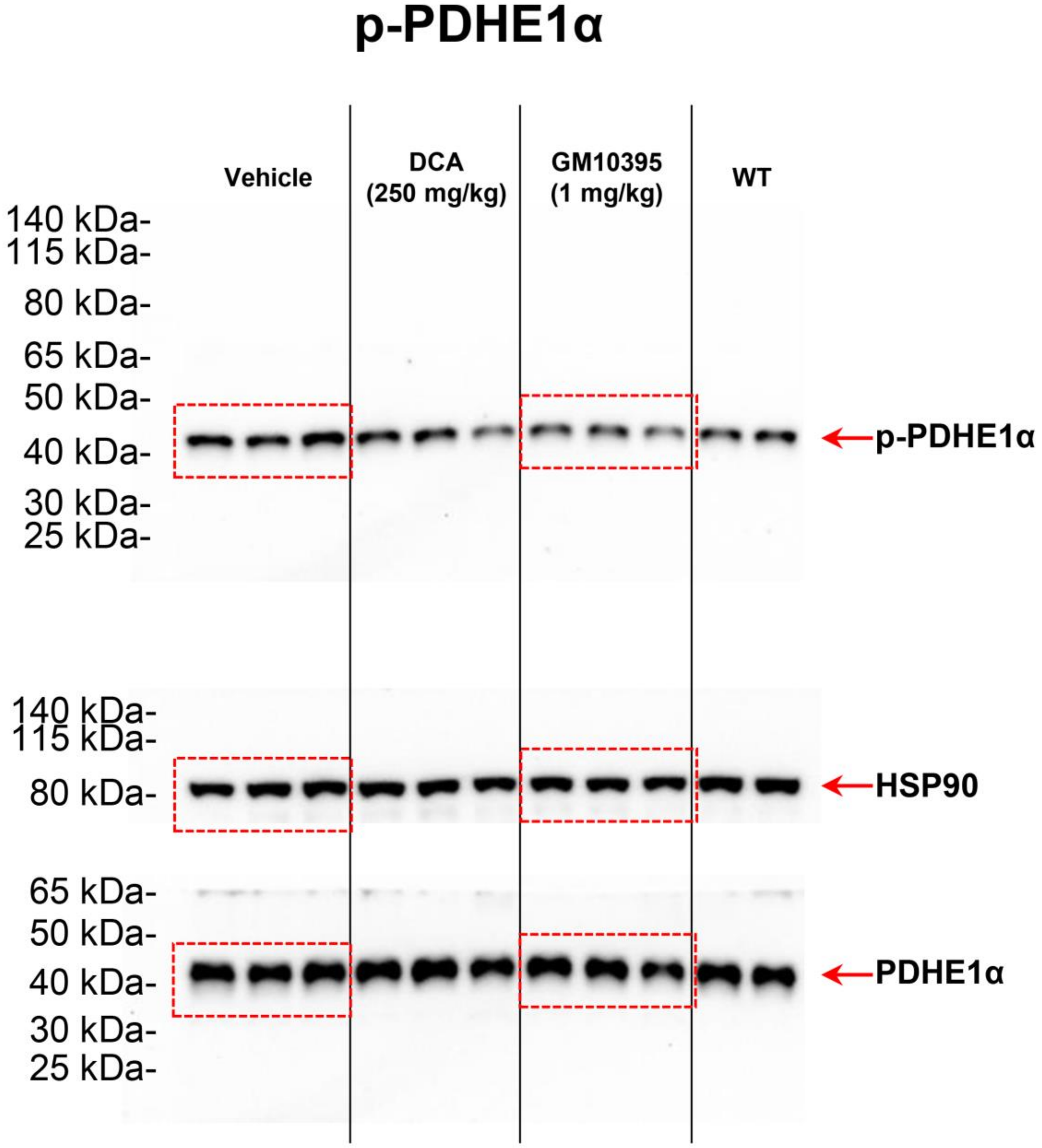

Full uncropped immunoblots of p-PDHE1α, PDHE1α and HSP90 from **Fig. 5C.** The red-dotted rectangle indicates the portion of the blot displayed in the manuscript.

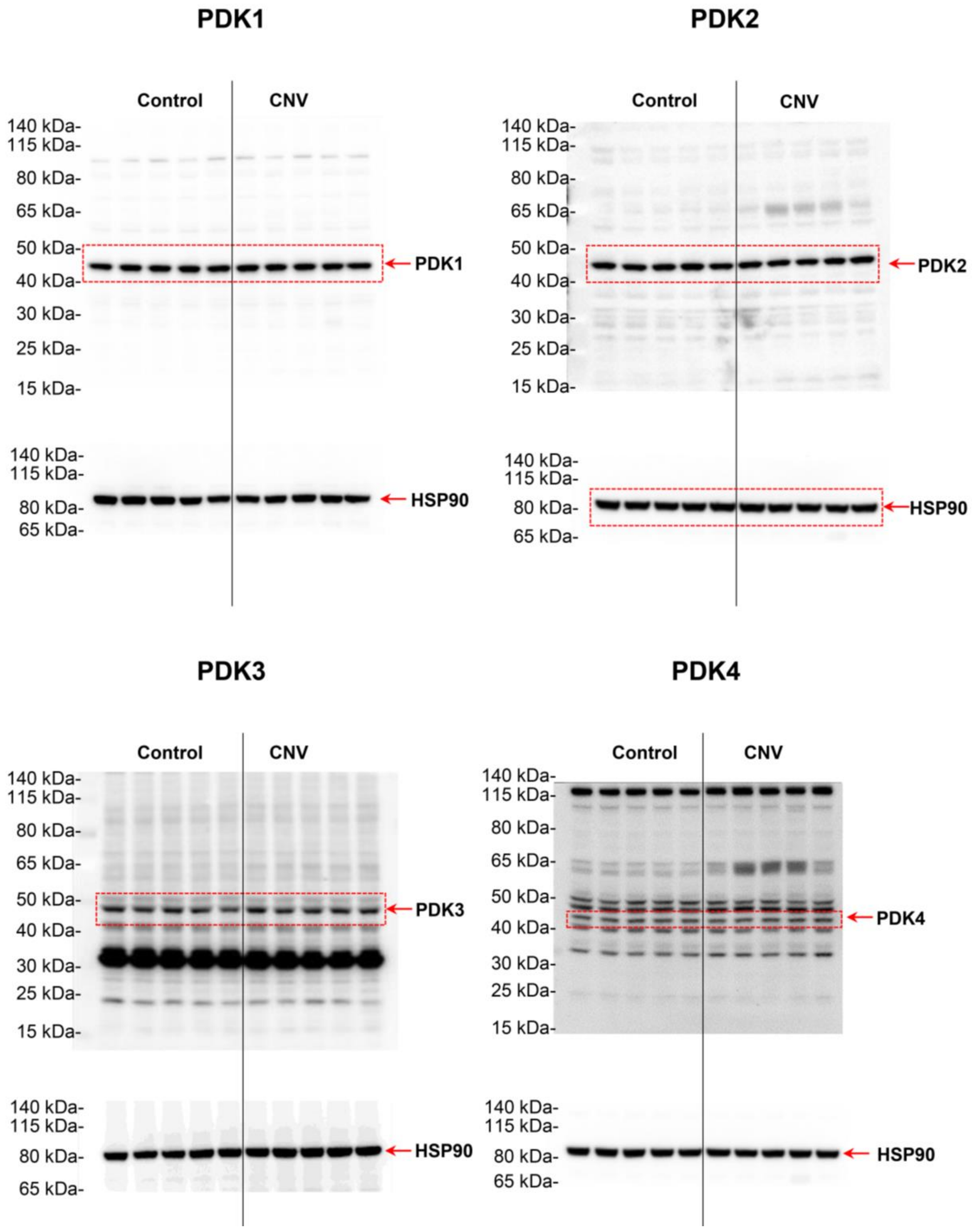

Full uncropped immunoblots of PDK1, PDK2, PDK3, PDK4 and HSP90 from **Fig. S1A.** The red-dotted rectangle indicates the portion of the blot displayed in the manuscript.
